# Factors Determining the Biodiversity of Halophilic Microorganisms on Historic Masonry Buildings

**DOI:** 10.1264/jsme2.ME16159

**Published:** 2017-06-08

**Authors:** Anna Otlewska, Justyna Adamiak, Teresa Stryszewska, Stanisław Kańka, Beata Gutarowska

**Affiliations:** 1 Institute of Fermentation Technology and Microbiology, Faculty of Biotechnology and Food Sciences, Lodz University of TechnologyWolczanska St. 171/173, 90–924 LodzPoland; 2 Institute of Building Materials and Structures, Faculty of Civil Engineering, Cracow University of TechnologyWarszawska St. 24, 31–155 CracowPoland

**Keywords:** halophilic microorganisms, historic masonry building, mineral-based materials, salt efflorescence, salinity

## Abstract

The aim of the present study was to obtain insights into the relationship between the chemical (salt content and pH) and physico-mechanical (humidity and compressive strength) properties of mineral-based materials from historic buildings with salt efflorescence and the growth and biodiversity of halophilic microorganisms. Samples were mainly characterized by pH 6.5–8.5 and a moisture content of between 0.12 and 3.3%. Significant variations were also found in the salt content (sulfates, chlorides, and nitrates) of the materials. An SEM/EDS analysis of material surfaces revealed the presence of halite, calcite, gypsum, sodium sulfate, and potassium-sodium sulfate. Culture-dependent and culture-independent (clone library construction) approaches were both applied to detect halophilic microorganisms. Results derived from culturable methods and the materials analysis revealed a correlation between the total halophile count and pH value as well as sulfate content. A correlation was not observed between the concentration of chlorides or nitrates and the number of halophilic microorganisms. The materials studied were inhabited by the culturable halophilic bacteria *Halobacillus* sp., *Virgibacillus* sp., and *Marinococcus* sp. as well as the yeast *Sterigmatomyces* sp., which was isolated for the first time from mineral materials. Culture-independent techniques revealed the following bacterial species: *Salinibacterium*, *Salinisphaera*, *Rubrobacter*, *Rubricoccus*, *Halomonas*, *Halorhodospira*, *Solirubrobacter*, *Salinicoccus*, and *Salinibacter*. Biodiversity was the highest in materials with high or moderate salinity.

Historic masonry buildings are constantly exposed to physical, chemical, and biological factors under severe climatic conditions, and are affected by different types of water impact such as rainwater and rising damp. The main component of masonry structures is porous brick containing various crystalline phases including quartz, albite, microcline, mullite, anorthoclase, hematite, halloysite, and mica as well as amorphous glassy phases. The composition of bricks varies and depends on raw materials and technological processes, particularly the firing temperature (5). In historic buildings, bricks undergo degradation as a result of frost weathering and salt crystallization, which affect porosity and the contents of sulfates, chlorides, and nitrates, particularly in the surface layers. The other component of masonry structures is mortar. Mortar is most often lime-based in historic buildings (it was gradually superseded by cement binders). The primary constituents of mortar are calcite (calcium carbonate), the C-S-H phase (CaO·SiO_2_·H_2_O, hydrated calcium silicates), and quartz sand. Over time, the corrosive impact of the external environment leads to the formation of water-soluble products (both highly and sparingly soluble), such as calcium sulfate (gypsum). Due to its high alkalinity (pH 12.5), mortar is generally an unfavorable environment for the growth of microorganisms; however, following corrosive environmental processes, pH markedly decreases, creating optimum levels for microorganism development (33).

Salt efflorescence appears to be widespread in historic buildings. Locally concentrated hygroscopic salts as well as those present across porous building materials migrate in water through capillary action, dry out, and precipitate on the surface, eventually forming salt deposits (11). Salt precipitation is a serious threat to the structure of building materials because salt occupying larger pores produces very high pressure, resulting in cracking, powdering, flaking, and material loss (30). Two different groups of efflorescence can be singled out: primary efflorescence, which occurs markedly quicker as a result of weather conditions (ambient temperatures, humidity, and wind), and secondary efflorescence, which is generally based on an alternate moisture source and longer drying and re-wetting cycles. In most cases, the salt crusts covering wall surfaces consist not only of halite, but also of epsomite, gypsum, nitrates, and many other compounds. Salt crystallization is a serious problem that affects building materials such as mortar, slurry, brick, and paint coatings. Salt may occur in the material as a result of co-migration with water via capillary action (1).

The salty deposits on the surfaces of historic buildings offer suitable saline niches that appear to promote the growth and proliferation of halophilic microorganisms (11, 21, 39). Halophilic microbial communities are responsible for the formation of colored stains including white spots and rosy discolorations on the surface of building materials (20, 31). Their growth also contributes to structural destabilization, which, in conjunction with water impact, leads to physical stress on the materials. The most prevalent of these microorganisms are bacteria, represented by *Gammaproteobacteria* (*Idiomarina* spp., *Salinisphaera* spp., *Halomonas* spp.), *Firmicutes* (*Halobacillus* spp., *Bacillus* spp.), *Actinobacteria* (*Rubrobacter* spp.), and *Archaea* (*Halococcus* spp. and *Halobacterium* spp.). In contrast, salty environments generally inhibit the growth of fungi (with the exception of some, for example, *Wallemia* sp., *Eurotium* sp.) (30).

The considerable capacity of halophilic microorganisms to inhabit salt efflorescence cannot be neglected, and appropriate restoration strategies need to be implemented in order to prevent microbial growth and protect cultural heritage. Although previous studies focused on the types of halophilic microorganisms in the masonry of historic buildings (11, 21, 27, 31), there is still a lack of research on the factors influencing their growth and biodiversity. Therefore, it currently remains unclear whether relationship exists between environmental conditions, material types and characteristics (humidity, mechanical strength, and chemical properties), the growth of halophilic microorganisms, and the impact they have on the surface of building materials. The paper addresses this issue in the absence of previous research in this area.

The objective of the present study was to investigate whether the chemical and physico-mechanical properties of mineral materials influence the presence, biodiversity, and proliferation of halophilic microorganisms in historic buildings. Within this framework, we deal with the central question of whether salt efflorescence on historic materials constitutes a suitable environment for the development of halophilic microorganisms.

This study involved the following steps: [1] The chemical and physico-mechanical characterization of historic materials, including pH, moisture content, compressive strength, and the contents of chlorides, nitrates, and sulfates; [2] Examining the effects of the salt contents of the materials on the growth and biodiversity of halophilic microorganisms.

## Materials and Methods

### Site description and sampling

**Fig. 1:**
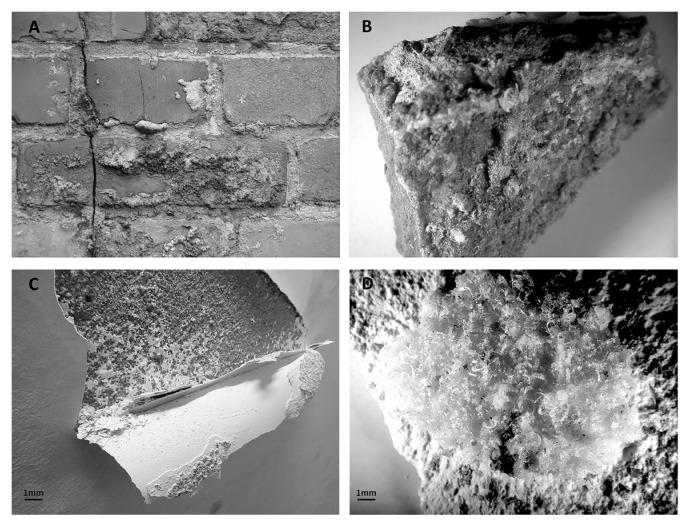
Efflorescence on the outside wall of a sedimentation tank (B150) (A); Stereomicroscopic images of a brick sample (sample B5) with visible symptoms of salt efflorescence on the surface (B); Detachment of paint coating due to salt efflorescence on the wall of a 19^th^ century building (C); Stereomicroscopic images of paint-coated plaster (sample P1) with visible symptoms of salt efflorescence on the surface (D).

Fifty samples of mineral material from the masonry of historic buildings: brick (*n*=34), mortar (*n*=12), and paint-coated plaster (*n*=4), were collected. The buildings were barracks no. B-114, B-124, and B-138, and trickling filters (B-150) located in the former Auschwitz II-Birkenau concentration and extermination camp in Brzezinka, and a 19th century historic townhouse in Lodz, Poland. Samples of materials were taken from internal and external structural elements in the form of drilled cores (for chemical and physicomechanical analyses), and directly from salt efflorescence (Fig. 1) by scraping with sterilized scalpels or spatulas (a microbiological analysis). Samples were collected between March and November 2015.

Chemical (sulfate, chloride, and nitrate contents and pH) and physical testing (humidity) were conducted on the 50 samples collected. Due to the historic importance of the buildings, the number of large samples (drilled cores with a diameter of 100 mm) was markedly limited and collected following consultations with the monument conservator. Thus, we could only collect 10 samples to assess the compressive strength of walls.

A microbiological analysis of mineral materials, based on culture-dependent methods, was conducted for 11 samples comprising brick (*n*=7) and paint-coated plaster (*n*=4). These samples were selected by analyzing salt contents according to the salt content classification criteria in Table 1. The samples tested exhibited low (*n*=2), medium (*n*=5), and high (*n*=4) salt contents. A description of the samples (B1–B7; P1–P4) used in the microbial analysis is given in Table 2.

The diversity of non-culturable halophilic bacteria was tested in four samples (B1, B3, B6, and P1) characterized by a low to high salt content and evaluated by the construction of 16S rRNA clone libraries (Table 2).

### Determination of the moisture content of building materials

The moisture content of samples was determined by weight loss on drying to a constant weight at 105°C and calculated according to the formula:

(1) $$\text{W}=({\text{m}}_{\text{w}}-{\text{m}}_{\text{s}})/{\text{m}}_{\text{s}}\times 100\;[\%]$$

where W is the moisture content, m_w_ is the sample weight upon collection, and m_s_ is the sample weight after drying to a constant weight.

### Determination of the compressive strength of walls

The compressive strength of samples was determined by the Z100 universal tester (Zwick/Roell) with an electromechanical drive unit using a nominal force of 100 kN. Samples were cylindrical cores with a diameter of 100 mm and a length corresponding to the thickness of the walls (approx. 120 mm) (15). Samples were mounted in the universal tester in a manner that reflected their original orientation within the wall and loaded to failure. The compressive strength of walls (f_c_^M^) was assessed according to the formula:

(2) $${{\text{f}}_{\text{c}}}^{\text{M}}=\text{q}\times {\text{F}}_{\text{ult}}/(\emptyset \times \text{l})\;[\text{MPa}]$$

where q is the correlation coefficient equal to 1.8, F_ult_ is the breaking force, Ø is the diameter of the cylindrical sample, and l is the length of the cylindrical sample.

### Chemical testing of mineral materials

Chemical tests included the assessment of pH and contents of sulfates, chlorides, and nitrates in core samples of mineral materials. They were ground in a ball mill (Fritsch) to a grain size less than 0.25 mm. Aqueous extracts were subsequently made at a 1:5 ratio of ground material to distilled water. The presence of sulfates, chlorides, and nitrates was examined spectrophotometrically using an AL800 spectrometer (AQUALYTICA). The concentrations of chloride, sulfate, and nitrate ions were measured using certified test kits from Tintometer GmbH (kits no. 419031, 532160, and 4530590). The results obtained were expressed as a percentage of the weight of the entire sample. The pH of the aqueous extracts was measured using a digital pH-meter (EUTECH CyberComm 6000). The salt content of the mineral materials was classified based on the Wissenschaftlich-Technische Arbeitsgemeinschaft für Bauwerkserhaltung und Denkmalpflege (45) criteria given in Table 1. In each case, the salt content level of the sample was assessed based on the salt type at the highest concentration.

### SEM/EDS microstructure examination

Samples were analyzed stereomicroscopically using a Discovery V20 microscope (Zeiss). The microstructure was examined using an EVO MA10 scanning electron microscope (Zeiss) equipped with an EDS XFlash 6/30 microanalyzer (Bruker). SEM/EDS images of the materials were obtained based on the detection of secondary electrons with a VPSE detector. Tests were performed on uncoated samples, prepared in the form of fracture surfaces. An SEM/EDS analysis was conducted in order to distinguish the salt types present in material samples.

### Culture-dependent analysis of halophilic microorganisms

The microbiological colonization of historic mineral materials was assessed using the plate count method. One gram of mineral material was suspended in saline (0.85%). The suspensions were plated on DSMZ medium 372 (20% [w/v] NaCl; 2% [w/v] MgSO_4_×7H_2_O) in order to examine the total count of halophilic microorganisms (11). Plates were incubated at 25±0.5°C for 30 d. The results obtained are expressed in CFU g^−1^ of material. All experiments were performed in triplicate.

### Molecular identification of isolated halophiles

Chromosomal DNA was extracted using the Genomic Mini Kit (A&A Biotechnology, Gdynia, Poland), according to the manufacturer’s instructions. The amplification of the bacterial 16S rRNA gene was performed with the universal primer pair 27f and 1492r (25). In the identification of yeast strains, fragments of approximately 500–600 bp, corresponding in size to the ITS1, ITS2 region and the 5.8 ribosomal RNA gene, were amplified with the primer set ITS1 and ITS4 (44).

All PCR reactions were conducted using the MJ Mini Gradient Thermal Cycler (Bio-Rad, Hercules, CA, USA) in a mixture containing 40 pmol of each primer, 1.5 U of RedTaq ReadyMix DNA polymerase (Sigma-Aldrich, St. Louis, MO, USA), and 20 ng of template DNA diluted to 50 μL with DNase-free water. PCR products were detected by electrophoresis in 1% agarose gel (w/v) in 0.5×TBE buffer (Sigma-Aldrich, St. Louis, MO, USA) and purified using the Clean-Up Mini Kit (A&A Biotechnology, Gdynia, Poland) following the manufacturer’s protocol.

### DNA extraction directly from mineral materials

Mineral samples were ground under liquid nitrogen using a mortar and pestle. DNA was extracted from 0.2–0.5 g of powdered samples employing FastDNA Spin Kit for Soil (MP Biomedicals, Solon, OH, USA). Extracted DNA was further purified with the QIAamp Viral RNA Mini Kit (Qiagen, Hilden, Germany).

### Construction and screening of 16S rRNA clone libraries

Bacterial 16S rRNA gene fragments (~585 bp) were amplified using the universal primer set 341f and 907r under the following thermal conditions: initial denaturation at 94°C for 2 min, 35 cycles consisting of denaturation at 94°C for 1 min, primer annealing at 47°C for 1 min and elongation at 72°C for 2 min, and a final extension step at 72°C for 2 min (25).

PCR products were ligated into the pGEM-T Easy Vector System (Promega, Mannheim, Germany) following the manufacturer’s protocol. Ligation products were transformed into *E. coli* JM109 and plated on LB medium containing ampicillin (100 μg mL^−1^), S-Gal (300 μg mL^−1^), and IPTG (0.1 mM). The screening of clone libraries was performed as described by Otlewska *et al.* (26).

The microorganisms identified were described as “halophiles” based on established knowledge and up-to-date literature (2, 11, 16, 19, 26, 31, 46).

### Sequencing of 16S rRNA and sequence analyses

All nucleotide sequences were obtained using the BigDye Terminator Ready Reaction Cycle Sequencing kit (Applied Biosystems, Foster City, CA, USA) and analyzed using an Applied Biosystems model 3730 Genetic Analyzer (Genomed S.A., Warsaw, Poland). The nucleotide sequences obtained from 103 clones (B1=27 clones, B3=29, B6=25; P1=22) were proofread, assembled, and aligned in Vector NTI Express Software (Life Technologies, Thermo Fisher Scientific, Waltham, MA, USA) and then compared with sequences available in The National Center for Biotechnology Information (NCBI, Bethesda, MD, USA) database using the blastn algorithm (BLASTN 2.2.30+) (47).

The nucleotide sequences obtained in this work were deposited to GenBank NCBI under the following accession numbers KU550578–KU550585.

### Multivariate data analysis

The relationship between material parameters (sulfate, chloride, and nitrate contents, pH) and the growth of halophilic microorganisms was characterized by Pearson’s correlation coefficients using Statistica v. 10.0 software (Stat Soft., USA). The level of significance was set at *p*<0.05. The strength of the correlation was examined according to Hinkle *et al.* (17): 0.9 to 1.0 (−0.9 to −1.0)—very strong positive (negative) correlation; 0.7 to 0.9 (−0.7 to −0.9)— strong positive (negative) correlation; 0.5 to 0.7 (−0.5 to −0.7)— moderate positive (negative) correlation; 0.3 to 0.5 (−0.3 to −0.5)— weak positive (negative) correlation; 0.0 to 0.3 (0.0 to −0.3)—negligible correlation.

## Results and Discussion

### Chemical and physico-mechanical characterization of historic mineral materials

An evaluation of the chemical and physico-mechanical properties of historic brick and paint-coated plaster was conducted in order to assess the extent to which they provide suitable conditions for the growth and proliferation of halophilic microorganisms.

**Fig. 2:**
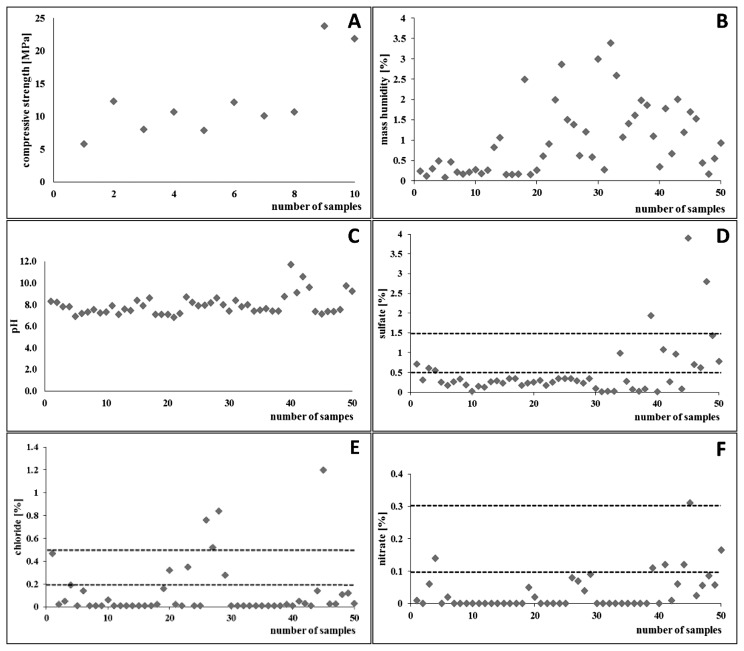
Physico-mechanical and chemical features of mineral materials. Compressive strength (A); Mass humidity (B); pH value of materials (C); Sulfate content (D); Chloride content (E); Nitrate content (F). The range of salt levels in materials according to WTA (45) is marked with the dashed line.

Compressive strength results markedly varied between 5.8 and 23.8 MPa (Fig. 2A). However, since compressive strength was assessed for samples cut from wall segments, which were sufficiently well preserved, it was assumed that there are numerous wall fragments with lower compressive strengths in the buildings studied than that given above. This variation in compressive strength is primarily attributable to differences in brick strength rather than mortar strength, which plays a lesser role. A close relationship exists between compressive strength and moisture and salts content in samples. Excessive dampness reduces compressive strength as a result of the partial dissolution of bonds between crystals in the structural lattice. Moreover, according to Foraboschi and Vanin (14), compressive strength may be lower or higher in the presence of salts, than in samples without salt, depending on the moisture content.

The moisture content of the mineral materials studied varied between 0.12% and 3.3% during summer (Fig. 2B), and did not exceed 3% in most samples. A possible explanation for the low moisture content is the low thickness (approx. 12 cm) of the walls, which may dry quickly after periods of dampness. A previous study conducted by Piotrowska *et al.* (32), in the former Auschwitz-Birkenau concentration and extermination camp, showed that the moisture content of mineral materials increased to 8–20% during autumn and winter (depending on the building location and height from the ground), leading to very damp or wet walls. Excessive dampness adversely affects compressive forces, while, as a result of abrupt changes in the moisture content, individual components gradually dehydrate leading to very high contracting stresses and destruction of the material by cracking, flaking, and plaster peeling. In addition, moisture content fluctuations promote the migration of water containing dissolved salts towards the surface of the material, providing good conditions for the growth of halophilic microorganisms (12, 23).

From the point of view of the present study, pH is a very important characteristic of mineral-based materials for the growth of halophiles. The optimal pH range for halophiles cultivated under laboratory conditions was previously reported to be between 6.0–7.0 (3). Among the materials tested, the pH of mortar and brick was similar; more than 80% of the samples had a pH between 6.8 and 8.5 (Fig. 2C). The low pH of mortar in the tested buildings was attributable to the poor state of the buildings, and the conditions under which they had been used. Only 8 out of the 50 tested samples had a pH of more than 8.5 (8.7–11.7). Environments with neutral pH enable the growth and proliferation of halophilic microorganisms (2), which suggests that the materials studied are suitable for their development. It is important to note that the pH of mortar is typically markedly higher, exceeding 11. According to Tiano *et al.* (42), pH is the main factor inhibiting microbial growth on mineral substrates. Moreover, mineral materials corrode through the leaching of alkaline oxides into solution, which may also increase the pH.

One of the critical characteristics of mineral materials is the salt content, which is determined by the concentrations of sulfates, chlorides, and nitrates. The sulfate content in 75% of the samples tested did not exceed 0.5% by weight (defined as a low salt content according to the criteria specified in the WTA guidelines), in 20% of the samples it ranged between 0.55% and 1.43% (defined as a medium salt content), and only 3 samples exhibited a high sulfate content (between 1.9% and 3.9%) (Fig. 2D).

In most of the samples tested (*n*=43), the content of chloride ions was low (less than 0.2% by weight). The other 20% of samples exhibited medium and high chloride contents (0.28–0.47% and 0.52–1.20%) (Fig. 2E). The nitrate content was generally the lowest; it did not exceed 0.1% by weight (low salt content) in 90% of samples, 8% of the samples had a medium nitrate content (0.09–0.17%), while only one sample had a high nitrate content (0.31%) (Fig. 2F).

Water-soluble salts accumulate in walls as a result of the transport of water by capillary action from the ground. These salts may crystallize on the surfaces of porous media as efflorescence or inside as subflorescence (9). Efflorescence appears as thin, foggy, and mostly white salt deposits on the surface of porous building materials. They not only present an esthetic issue, but may also severely damage the microstructure of the material (4, 6). The presence of salts correlates with the durability of masonry elements. The greatest risk is posed by sodium and potassium sulfates, and, to a lesser extent, by calcium sulfates. These compounds crystallize at different hydration levels, which causes changes in the volume of salt crystals, and results in strong crystallization pressure in the subsurface region of the wall. The resulting tensile stress has a destructive effect on the wall (10). In extreme cases of a very high salt content, salts may substantially lower the mechanical properties of bricks and mortar. Moreover, they hinder the process of wall drying, contributing to a permanently higher moisture content in buildings (41). In contrast, in an environment with low relative humidity, bricks that do not contain salt may dry completely.

### Salt characterization in mineral samples from historic materials

**Fig. 3:**
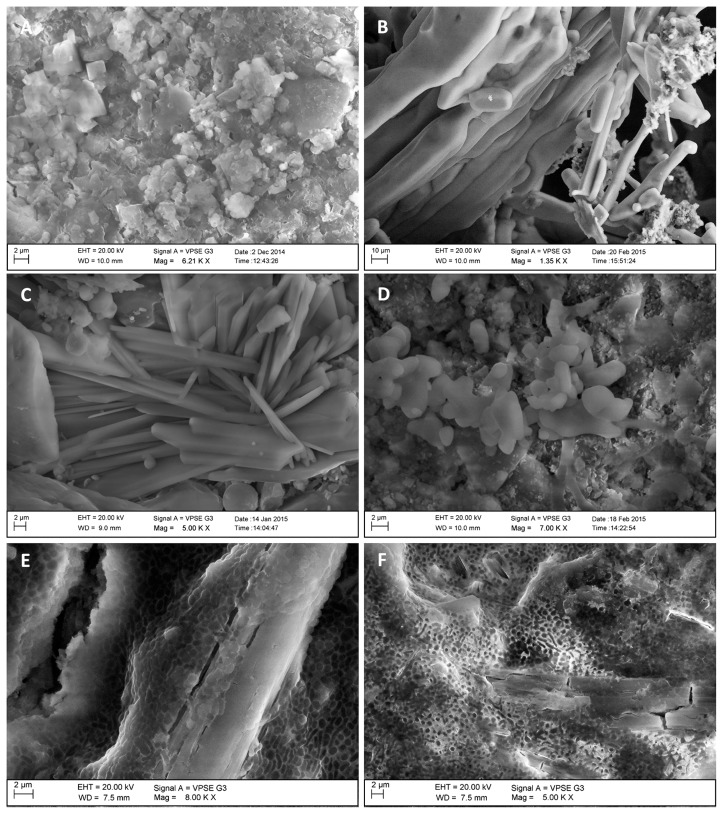
Crystals of sodium chloride on brick sample B3 (element content according to an EDS analysis: Chlorine 9.7–10.9, Sodium 7.2–12.5 wt%) (A); Crystals of sodium chloride on paint-coated plaster P4 (Chlorine 36.0–41.5, Sodium 29.0–32.4 wt%) (B); Crystals of calcium and potassium sulfates on brick sample B5 (Oxygen 39.4–53.4, Sulfur 10.8–14.8, Calcium 11.9–20.6, Potassium 2.5–11.4 wt%) (C); Crystals of sodium sulfate on plaster-coated paint sample P1 (Oxygen 41.8–45.1, Sulfur 7.3–7.5, Sodium 10.3–15.6 wt%) (D); Microorganism cells on sulfate crystals (E); Microorganisms cells on chloride crystals (F).

**Fig. 4:**
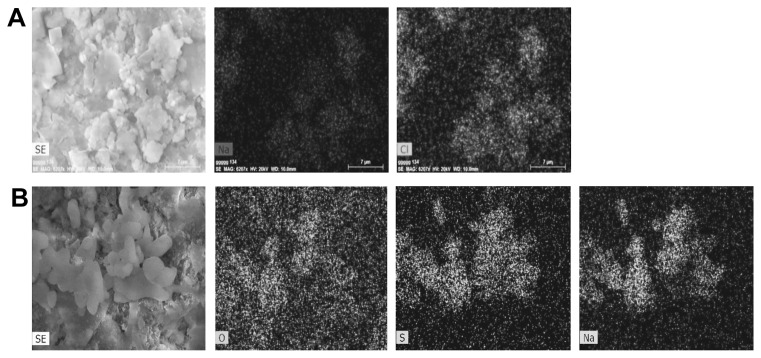
The distribution of elements based on an SEM/EDS analysis. (A) Brick sample B3—the presence of sodium chloride; (B) paint-coated plaster sample P1—the presence of sodium sulfate.

Historic mineral materials with symptoms of deterioration were found to contain various hygroscopic salts, such as chlorides, nitrates, and sulfates, near the surface. An EDS analysis of these surfaces showed that the chemical composition of salt efflorescence, within the buildings of the former Auschwitz-Birkenau concentration and extermination camp, varied. Calcium sulfate (CaSO_4_, gypsum), calcium carbonate (CaCO_3_, calcite), and sodium chloride (NaCl, halite) were found on bricks inside the buildings (Fig. 3A and 4A), while the predominant salt in the brick samples from external parts of the buildings (*e.g.*, sedimentation tank, B-150) was potassium-calcium sulfate (Fig. 3C). The mineralogical composition of samples from the façade of the 19th century townhouse was characterized by a high gypsum content, while the powdery material from the internal, paint-coated walls varied and contained, in addition to gypsum, halite (Fig. 3B), calcite, and sodium sulfate (Na_2_SO_4_) (Fig. 3D and 4B). However, it is important to note that efflorescence is not uniformly distributed in the subsurface region. Salt deposits are inhomogeneous as a result of water circulation in the material—repeated wetting and drying cycles caused by humidity and temperature fluctuations (39). Apart from salts, EDS revealed the presence of other chemical compounds typical of mineral building materials, including SiO_2_, Al_2_O_3_, and Fe_2_O_3_.

Soluble salts, such as gypsum, halite, calcite, sodium sulfate, and potassium-sodium sulfate, found in the tested samples, are widespread components of efflorescence in historic buildings. Laiz (21) and Piñar *et al.* (29, 27) reported high amounts of halite, calcite, and gypsum in efflorescence on the walls of the Cathedral of Jerez (Spain), the Chapel of Castle Herberstein (Austria), and the 14th century Chapel of St. Virgil (Austria). High salt and water contents and a mainly neutral pH create a unique microclimate on the surface of historic buildings, enabling the growth of various halophilic microorganisms, the cells of which were observed during the SEM/EDS analysis (Fig. 3E and F).

However, it currently remains unclear whether marked variations in the chemical compositions of salt efflorescence as well as spatial and temporal changes are conducive to the growth and proliferation of halophilic microorganisms. In order to clarify this issue, samples of mineral materials were subjected to a microbiological analysis.

### Effects of the salt content on the growth of halophilic microorganisms

**Fig. 5:**
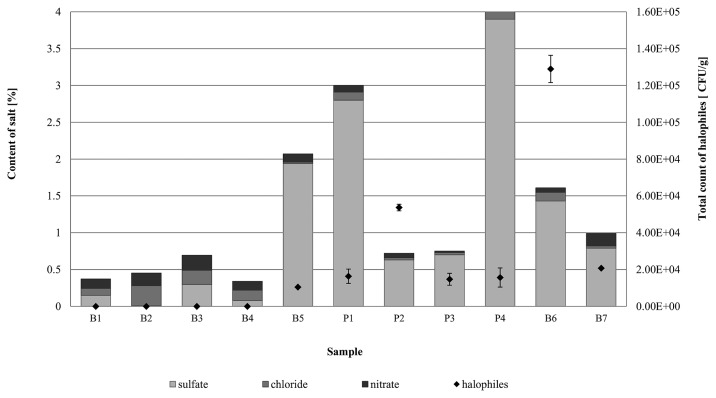
Total count of halophilic microorganisms [CFU g^−1^] in tested mineral materials (B—brick; P—paint coated plaster) in relation to nitrate, chloride, and sulfate contents [%].

Samples (B1–B4) from construction elements inside the buildings of the former Auschwitz II-Birkenau concentration and extermination camp had low sulfate (0.008–0.298%) and chloride (0.096–0.192%) contents and medium nitrate levels (0.12–0.20%). In these samples, no halophilic microorganisms were detected using culture-dependent methods (Fig. 5). The exception was the brick sample from the sedimentation tank (building B150, sample B5), in which the number of halophilic microorganisms was high and equaled 3.1×10^4^ CFU g^−1^. This particular sample exhibited high sulfate (1.94% by weight), but low chloride (0.02%) and nitrate (0.11%) concentrations (Fig. 5). Nevertheless, the sedimentation tank differed from the other historic buildings studied because it is an open structure without a roof, the technical condition of which is influenced by variable environmental conditions (temperature, precipitation, insolation, and air humidity) and its location (near two rivers). Cyclical changes in air temperature and the capillary movement of water cause a lot of salt efflorescence on the brick surface, providing an environment conducive to the growth of halophilic microorganisms.

A high content of SO_4_^2−^ ions in the mineral materials (0.7–3.9%) was also found in samples collected from the internal walls (P1–P3) and façade of the 19th century townhouse (B6–B7). Irrespective of the type of material, the total count of halophilic microorganisms for these samples was markedly higher than that for samples from the former Auschwitz II-Birkenau concentration and extermination camp. The number of halophiles in paint-coated plaster from inside the townhouse ranged between 1.4×10^4^ and 1.6×10^5^ CFU g^−1^ (P1–P3), and between 2.1×10^4^ and 1.3×10^5^ CFU g^−1^ (B6–B7) for bricks from the external façade (Fig. 5).

In culture-based experiments, relationships between salinity levels (sulfate, chloride, and nitrate contents), the pH of mineral materials, and the growth of halotolerant/halophilic microorganisms were examined basing on Pearson’s correlation coefficients. The total count of halophiles and sulfate ion concentration revealed a positive and moderate correlation (*r*=0.46; *p*<0.05; *N*=11). On the other hand, microorganism growth appeared to negatively correlate with the chloride content; however, this relationship was negligible (*r*=0.17; *p*<0.05; *N*=11). A negative, but moderate correlation was also observed between the number of halophiles and nitrate levels in the tested mineral sample (*r*=0.41; *p*<0.05; *N*=11). pH was another factor that positively and moderately correlated (*r*=0.40; *p*<0.05; *N*=11) with the number of halophiles.

The positive correlation in terms of the sulfate content and halophile growth needs to be highlighted. Halophilic microorganisms were mainly detected in samples containing high concentrations of SO_4_^2−^ ions. Therefore, the presence of these microorganisms may be influenced by the sulfate content. According to Schneegurt (40), sulfate often appears as a counterion for Ca, Mg, Fe, Mn, N, and K salts. Research has focused on the role of chloride for growth stimulation, endospore germination, the synthesis of flagellin (a major component of the flagellum), motility, and the transport of the compatible solute glycine betaine of moderately halophilic bacteria (8, 35, 36, 38). The latter of these functions is closely connected to osmolytes and osmoadaptation. Halophiles exhibit two mechanisms of adaptation to life in a high salt environment. One of these mechanisms, termed “salt in cytoplasm”, involves the accumulation of inorganic salts (K^+^ or, more rarely, Na^+^ cations and Cl^−^ anions) in the cytoplasm. Chloride ions are transported via a light-dependent cell machinery with halorhodopsin or energized by symport with Na^+^ (24). The other mechanism is based on the synthesis of compatible solutes, such as ectoine, proline, and glycine betaine. According to Roeßler and Müller (37), in the case of some bacteria (*e.g.*, *Halobacillus halophilus*), the uptake of glycine betaine under steady state conditions and after osmotic upshock is Cl^−^-dependent.

### Phylogenetic analysis and biodiversity of the halophilic community

Thirteen genera of bacteria and one genus of yeast were detected in samples using culture-dependent and independent methods (Table 3). The degree of similarity of all analyzed nucleotide sequences ranged between 94 and 100%.

Culturable bacteria belonging to the phylum *Firmicutes* were detected and isolated from brick samples (B1–B7). Most halotolerant/halophilic isolates (50%) were closely affiliated to *Halobacillus* spp. (*H. naozhouensis*, *H. hunanensis*, and *H. styriensis*), while the remaining halotolerant/halophilic bacteria were related to *Marinococcus halophilus* and *Staphylococcus succinus*. *Halobacillus* spp. is generally the most frequently isolated microorganism from historic buildings with salt efflorescence. In their study of salt-damaged medieval wall paintings and building materials from Herberstein Castle in St. Johann bei Herberstein, Styria, Austria and St. Virgil Chapel in Vienna, Austria, Ripka *et al.* (34) found different species of *Halobacillus*. Moreover, *H. naozhouensis* and *H. styriensis* were isolated from exhibition areas of the Capuchin Catacombs in Palermo, Italy (31). The abundance of *Halobacillus* spp., moderately halophilic bacteria, in extreme conditions, such as salt efflorescence, indicates that they grow and proliferate under moderate to high salt concentrations. However, it is important to note that *Halobacillus* spp. are generally detected using culture-dependent methods, while the vast majority of halophilic bacteria may be detected using only molecular-based techniques (2, 30). The culture-independent analysis on brick samples (B1), with low salinity levels, not only identified members of the phylum *Firmicutes*, but also *Proteobacteria* and *Actinobacteria* members dominating the bacterial community. Amongst these three classes, halotolerant/halophilic species affiliated to *Halomonas*, *Rubrobacter*, and *Virgibacillus* were detected. *Proteobacteria*, *Actinobacteria*, and *Firmicutes* were the predominant phylogenetic groups in the brick (B3) characterized by moderate salinity, similar to the sample with lower salinity. The phylum *Proteobacteria* was represented by the halophilic species *Salinisphaera*, *Halorhodospira*, *Halomonas*, and *Marinobacter*. *Marinobacter* sp. and *Salinisphaera* sp. were the predominant species, representing 77% of all nucleotide sequences associated with halophiles. *Salinisphaera* is highly tolerant to salt contents, even up to 28% NaCl (w/v). Furthermore, all of the above-described species were previously detected on historic buildings with salt efflorescence (28). Among *Actinobacteria*, *Salinibacterium* sp. and *Rubrobacter* sp. were identified. Additionally, in this sample, clones belonging to the class *Bacteroidetes* were also identified and were mainly represented by *Rubricoccus* sp. Halophilic members of *Bacteroidetes* are most commonly isolated from different hypersaline environments including medieval frescoes in the Crypt of the Original Sin (Matera, Italy), ancient wall paintings of the Chapel of St. Virgil (Vienna, Austria), the Etruscan tomb, and the Capuchin Catacombs of Palermo (Italy) (7, 18, 29). The highest diversity of halophilic bacteria was observed in the brick sample with high salinity levels (B6). All the above-described genera were detected in this sample, in addition to *Salinibacter* sp. (*Bacteroidetes*), *Marinococcus* sp., and *Salinicoccus* sp. (*Firmicutes*) (Table 3).

Fewer species were detected in paint-coated plaster samples (P1, P2). The co-existence of the bacteria *Virgibacillus halodenitrificans* with the yeast *Sterigmatomyces halophilus* was observed among culturable halotolerant/halophilic microorganisms (Table 3). Of interest is the yeast *S. halophilus* being exclusively found on mineral samples (B5–B7 and P1–P3) with a medium or high content of sulfates (0.6–3.9% by weight), irrespective of sample pH. *S. halophilus* was isolated for the first time by Fell (13) from a marine environment and described as a non-filamentous yeast based on the formation of conidia on sterigmata. Previous studies (29, 43) on endo- and epilithic fungal communities colonizing historic buildings focused exclusively on the detection and identification of hyphomycetous fungi, disregarding yeasts. Also, little is known about the role of yeasts, whether halophilic or not, in their biodeterioration.

In addition, the culture-independent methods applied to paint-coating samples enabled the detection of only one member of the class *Actinobacteria*, related to *Rubrobacter* sp. (Table 3). This species was detected in all samples analyzed, regardless of the salt content and type of material. This phenomenon may be associated with the bacteria belonging to *Rubrobacter* species being a very different group. Some strains (*e.g.*, *R. radiotolerans*, *R. xylanophilus*, and *R. taiwanensis*) are slightly halotolerant because they grow at an NaCl (w/v) concentration of 5–6%; however, they also are able to grow in the absence of NaCl (29). It is interesting that these *Rubrobacter* strains require Na or Mg ions for growth, similar to moderate halophilic bacteria. In contrast, Jurado *et al.* (19) isolated an *R. bracarensis* strain from the interior walls of the Vilar de Frades Church (Portugal), which, in principle, requires an NaCl (w/v) concentration of 3–10% to grow, but were able to grow even at a concentration of 30%. As suggested by Laiz *et al.* (22), *Rubrobacter* play an active role in efflorescence niches and contribute to the biodeterioration processes of historic monuments. It is often associated with a rosy discoloration, leading to the degradation of monuments and irreversible damage to their appearance (11, 19).

Our results allowed halotolerant/halophilic microorganisms to be classified into 3 groups based on the salt content level (low, medium, and high) (Table 3). Most halotolerant/halophilic genera were detected in brick with a high salt concentration, while the fewest were present in samples characterized by low salinity. Among the halophiles identified, only four species: *Halobacillus*, *Halomonas*, *Rubrobacter*, and *Virgibacillus*, were common to all three sample types (low, medium, and high salt contents).

## Conclusions

The properties of historic mineral materials: salt content (chlorides, nitrates, and sulfates), humidity, pH, and periodic fluctuations, offer a unique microclimate for the growth of various halotolerant and halophilic microorganisms. The predominant cultivable microorganisms isolated from mineral materials were bacteria related to *Halobacillus* sp., *Virgibacillus* sp., and *Marinococcus* sp., and yeasts of the genus *Sterigmatomyces*. Importantly, the halophilic yeast *S. halophilus* was isolated and identified for the first time on mineral materials. Culture-independent techniques confirmed the presence of the isolated microorganisms, and also detected *Salinibacterium*, *Salinisphaera*, *Rubrobacter*, *Rubricoccus*, *Halomonas*, *Halorhodospira*, *Solirubrobacter*, *Salinicoccus*, and *Salinibacter* species. Our results showed that the highest biodiversity was found in materials characterized by high or moderate salinity.

## Figures and Tables

**Table 1:** Salt level of mineral materials (45)

Salt type	Salt content [% mass]		
chloride	<0.2	0.2–0.5	>0.5
nitrate	<0.1	0.1–0.3	>0.3
sulfate	<0.5	0.5–1.5	>1.5
Salinity level	Low	Medium	High

**Table 2:** Sampling sites and type of alteration

Sample number	Sampling area	Sampling site	Type of alteration	Type of material	Salinity level^B^	pH of the sample	Analysis^A^
B1	former Auschwitz II-Birkenau concentration and extermination camp in Brzezinka	floor; barrack B-124	Bulging, crumbling, cracking weak efflorescence	brick	low	7.36	C/NC/S
B2		partition wall, height from the ground 10 cm; barrack B-124	Bulging, crumbling, cracking	brick	medium	7.02	C/S
B3		partition wall, height from the ground 50 cm; barrack B-124	Bulging, crumbling, cracking	brick	medium	7.33	C/NC/S
B4		partition wall, height from the ground 50 cm; barrack B-138	Bulging, crumbling, cracking, weak efflorescence	brick	low	7.33	C/S
B5		external wall, height from the ground 50 cm; object B-150	Very strong efflorescence, cracking, detachment	brick	high	8.75	C/S

P1	19th century historic building in Lodz	internal, eastern load-bearing wall, height from the ground 150 cm	Strong efflorescence, cracking, detachment	paint-coated plaster	high	7.55	C/NC/S
P2		internal, eastern load-bearing wall, height from the ground 100 cm	Strong efflorescence, cracking, detachment	paint-coated plaster	medium	7.36	C/S
P3		internal, eastern partition wall, height from the ground 80 cm	Strong efflorescence, cracking, detachment	paint-coated plaster	medium	7.35	C/S
P4		internal, eastern partition wall, height from the ground 100 cm	Very strong efflorescence, powdering	paint-coated plaster	high	7.13	C/S
B6		external, western wall, height from the ground 50 cm	Strong efflorescence, detachment	brick	high	9.75	C/NC/S
B7		external, northern wall, height from the ground 80 cm	Very strong efflorescence, detachment	brick	medium	9.25	C/S

A Type of analysis: C—culture-dependent analysis (16S rRNA gene sequencing); NC—culture-independent analysis (clone library construction); S—SEM/EDS analysis

B salinity level according WTA (45) included in Table 1

**Table 3:** Detected halotolerant/halophilic microorganisms and their presence on historic mineral materials

Phylum	Number of clones	Closely related genus	Similarity (%)	Material type	Salinity level of halophile occurrence*	Inhabitance on historic mineral materials (found in the literature)
*Agaricostilbomycetidae*	nd	*Sterigmatomyces*^C^	100	brick (B5–B7) paint-coated plaster (P1, P3)	medium, high	nd

*Firmicutes*	nd	*Halobacillus*^C^	100	brick (B1–B7) paint-coated plaster (P4)	low, medium, high	wall paintings (34) catacombs (31)
	2	*Virgibacillus*^C/NC^	100/99	paint-coated plaster (P1–P2/P1)	low, medium, high	walls of well (46) catacombs (31)
	2	*Marinococcus*^C/NC^	100/95	brick (B6/B6)	high	wall paintings (11)
	4	*Staphylococcus* ^C/NC^	98/99	brick (B6–B7/B6)	medium, high	catacombs (31) wall paintings (11)
	2	*Salinicoccus*^NC^	96	brick (B6)	high	nd

*Proteobacteria*	8	*Salinisphaera*^NC^	96	brick (B3)	medium	catacombs (31) medieval wall painting (29)
	4	*Marinobacter*^NC^	99	brick (B3, B6)	medium, high	brick wall (16)
	3	*Halomonas*^NC^	96	brick (B1, B3, B6)	low, medium, high	catacombs (31) wall paintings (11)
	2	*Halorhodospira*^NC^	96	brick (B3)	medium, high	brick wall (11, 26)

*Actinobacteria*	2	*Rubrobacter*^NC^	99	brick (B1, B3, B6) paint-coated plaster (P1)	low, medium, high	wall paintings (19, 29)
	3					
	1	*Salinibacterium*^NC^	99	brick (B3, B6)	medium, high	brick wall (16, 26)

*Bacteroidetes*	1	*Salinibacter*^NC^	94	brick (B6)	high	nd
	2	*Rubricoccus*^NC^	98	brick (B3, B6)	medium, high	nd

nd—not detected

C microorganisms detected by the culture-dependent method (16S rRNA gene sequencing)

NC microorganisms detected by the culture-independent method (clone library construction)

* total concentration of chloride, sulfate, and nitrate
